# Supplementary material for: A high-resolution mRNA expression time course of embryonic development in zebrafish
Source: eLife. 2017 Nov 16;6:e30860. doi: 10.7554/eLife.30860 (PMC5690287; doi:10.7554/eLife.30860)
Supplement: Supplementary file 6. [file elife-30860-supp6.zip › biolayout-clusters-files/Cluster153.html]

Cluster153


# Cluster153: Detail

### Go to ZFA detail

## GO

| GO ID | Description | Domain | Annotated | Expected | Observed | Adjusted p-value | Genes | Ensembl IDs |
| --- | --- | --- | --- | --- | --- | --- | --- | --- |
| GO:0055114 | oxidation-reduction process | biological\_process | 343 | 0.32 | 5 | 0.011 | ywhah dldh pdha1a hadh cs | ENSDARG00000005560 ENSDARG00000008785 ENSDARG00000012387 ENSDARG00000030765 ENSDARG00000103364 |
